# Obesity and cardio-metabolic risk factors in urban adults of Benin: Relationship with socio-economic status, urbanisation, and lifestyle patterns

**DOI:** 10.1186/1471-2458-8-84

**Published:** 2008-03-04

**Authors:** Roger Sodjinou, Victoire Agueh, Benjamin Fayomi, Hélène Delisle

**Affiliations:** 1 TRANSNUT, WHO Collaborating Centre on Nutrition Changes and Development, Department of Nutrition, Faculty of Medicine, Université de Montréal, C.P. 6128 Succursale centre-ville, Montréal Qc H3C 3J7, Canada; 2 Institut Régional de Santé Publique, Route des Esclaves, 01BP918, Ouidah, Bénin; 3 Institut des Sciences Biomédicales Appliquées, Université d'Abomey-Calavi, 01BP188 Cotonou, Bénin

## Abstract

**Background:**

There is a dearth of information on diet-related chronic diseases in West Africa. This cross-sectional study assessed the rate of obesity and other cardiovascular disease (CVD) risk factors in a random sample of 200 urban adults in Benin and explored the associations between these factors and socio-economic status (SES), urbanisation as well as lifestyle patterns.

**Methods:**

Anthropometric parameters (height, weight and waist circumference), blood pressure, fasting plasma glucose, and serum lipids (HDL-cholesterol and triglycerides) were measured. WHO cut-offs were used to define CVD risk factors. Food intake and physical activity were assessed with three non-consecutive 24-hour recalls. Information on tobacco use and alcohol consumption was collected using a questionnaire. An overall lifestyle score (OLS) was created based on diet quality, alcohol consumption, smoking, and physical activity. A SES score was computed based on education, main occupation and household amenities (as proxy for income).

**Results:**

The most prevalent CVD risk factors were overall obesity (18%), abdominal obesity (32%), hypertension (23%), and low HDL-cholesterol (13%). Diabetes and hypertriglyceridemia were uncommon. The prevalence of overall obesity was roughly four times higher in women than in men (28 vs. 8%). After controlling for age and sex, the odds of obesity increased significantly with SES, while a longer exposure to the urban environment was associated with higher odds of hypertension. Of the single lifestyle factors examined, physical activity was the most strongly associated with several CVD risk factors. Logistic regression analyses revealed that the likelihood of obesity and hypertension decreased significantly as the OLS improved, while controlling for potential confounding factors.

**Conclusion:**

Our data show that obesity and cardio-metabolic risk factors are highly prevalent among urban adults in Benin, which calls for urgent measures to avert the rise of diet-related chronic diseases. People with higher SES and those with a longer exposure to the urban environment are priority target groups for interventions focusing on environmental risk factors that are amenable to change in this population. Lifestyle interventions would appear appropriate, with particular emphasis on physical activity.

## Background

The burden of cardiovascular disease (CVD) has increased over the last two decades in nearly all developing countries, particularly in urban areas [1,2]. According to WHO, about 80% of all deaths from CVD worldwide now occur in developing countries, and it is estimated that by the year 2010, CVD will be the leading cause of death in these countries [1]. A major factor of the increasing prevalence of CVD in developing countries is the on-going nutrition transition with progressive shifts to a westernized diet high in saturated fats and sugar, and a more sedentary lifestyle [3]. Urbanisation and globalisation are fuelling the nutrition transition. These changes result in rapidly increasing levels of obesity, type 2 diabetes, dyslipidemia and hypertension, collectively known as the metabolic syndrome.

The nutrition transition has been documented in some African countries, including South Africa [4] and Gambia [5]. To date, no such data are available for West Africa, and in Benin, for instance, the only indicative information is that overweight and obesity are increasing among adults [6]. Cotonou, the largest city of Benin, has urbanised rapidly over the last few decades [7] and this may facilitate the adoption of lifestyles that are conducive to obesity and cardio-metabolic risk factors [8]. Previous studies in Cameroon and in South Africa have shown that exposure to the urban environment is associated with increased risk of obesity, diabetes or hypertension [9,10] but this has to be verified in other settings. Moreover, metabolic diseases may not affect all segments of the population equally. Therefore, assessing the associations of CVD risk factors with SES, as well as urbanisation, would yield valuable information for policy and programmes.

Current scientific evidence suggests that the adoption of healthy lifestyles, i.e. balanced diet, increased physical activity, tobacco abstention (or avoidance), and moderate alcohol consumption can prevent and help control chronic diseases [11]. In the INTERHEART study, smoking, daily consumption of fruits and vegetables, regular alcohol consumption, regular physical activity, and other risk factors (history of hypertension, diabetes, abdominal obesity, psychosocial factors) were all significantly related to acute myocardial infarction [12]. Physical inactivity and smoking have been reported to contribute the most to the risk of chronic disease in the US, followed by dietary intake and alcohol use [11]. However, few studies have examined lifestyles and their relationship with risk of chronic diseases in sub-Saharan African countries, mainly because of the recent emergence of such diseases in the area [2]. Over the last decades, several studies have examined the relationship of chronic diseases with single lifestyle factors. Although this approach provides valuable information for public health interventions, it does not take into account the possible clustering of lifestyle factors and their combined effect over time on the risk of chronic disease. Combining lifestyle factors in a single composite measure, for example a lifestyle index as developed recently by Kim et al [11], can be an effective way of assessing the impact of lifestyles on the risk of chronic disease.

A cross-sectional study was conducted in Cotonou to assess the prevalence of obesity and cardio-metabolic risk factors in apparently healthy urban adults, and to explore the association of urbanisation status and SES with the risk factors. An additional objective was to investigate the association of several modifiable lifestyle factors with these risk factors. We hypothesized that obesity and cardio-metabolic risk factors were positively associated with SES and longer exposure to the urban environment. We also expected healthy lifestyle behaviours to be associated with a lower risk, particularly hypertension, which is widespread in sub-Saharan African populations [13].

## Methods

### Subjects

The study was conducted in the city of Cotonou, with an estimated population of one million inhabitants [14]. Eligible participants were born-Beninese adults aged 25 to 60 years who had been living in Cotonou for at least 6 months. Subjects with a prior history of hypertension, diabetes or coronary heart disease were excluded because their condition was likely to have altered their eating and lifestyle patterns. Pregnant and lactating women, as well as physically and mentally disabled subjects, were also excluded.

Cotonou is divided into 140 neighbourhoods of approximately equal population size. Ten neighbourhoods were picked at random. The administrative maps of the selected neighbourhoods allowed the random selection of 20 households in each of them. One subject was randomly selected among the eligible members of every household during home visits. We alternated men and women to have an equal number of subjects for each sex. Overall, 100 men and 100 women participated in the study. This sample size was deemed representative of the apparently healthy adult population of the city, and adequate to perform multivariate analyses with 20 independent variables and detect even small size effects, with 80% statistical power and a 95% confidence level [15].

### Anthropometric data

Body weight was measured on subjects in light clothing and without shoes to the nearest 0.1 kg using a mechanical scale (SECA^®^, Germany). Height was measured to the nearest 0.1 cm with a commercial stadiometer (SECA^®^, Germany). BMI was calculated as weight in kilograms divided by height in meters square. Overall obesity was defined as a BMI ≥ 30 [16]. Waist circumference was measured to the nearest 0.1 cm with a measuring tape at midpoint between the last rib and the iliac crest, with the subjects standing and breathing normally [17]. Abdominal obesity was defined as a waist circumference of ≥ 102 cm in men or ≥88 cm in women according to WHO [18].

### Blood pressure

Two readings of systolic and diastolic blood pressure were taken on the right arm of each subject in a sitting position after a 10-minute rest, using a mercury sphygmomanometer. The time interval between the first and the second reading was at least 20 minutes. The mean of the two readings was used in the analyses. Hypertension was defined as systolic blood pressure ≥ 140 mmHg and/or diastolic blood pressure ≥ 90 mmHg [19], and this applies to newly detected subjects.

### Biochemical analyses

Venous blood samples (10 ml) were drawn after an over-night fast of 12 hours and were centrifuged within two hours. Fasting plasma glucose was determined using the glucose oxidase method. Serum concentrations of high-density lipoprotein (HDL) cholesterol and triglycerides were analysed by enzymatic methods. For quality assurance of the analyses, certified laboratory standards from the USA were used. Diabetes was defined as fasting plasma glucose ≥7 mmol/l [18], and this applies to newly detected subjects. Low HDL cholesterol cut-offs were <0.9 mmol/l (for men) and <1 mmol/l (for women). Hypertriglyceridemia was defined as triglycerides ≥1.7 mmol/l [18].

### Lifestyle factors

An overall lifestyle score (OLS) was constructed based on four modifiable lifestyle factors that have been shown to influence the risk of chronic diseases: diet, smoking, alcohol consumption and physical activity [11]. A partial score was computed for each of these components.

#### Diet quality score (DQS)

Dietary intake was assessed through three non-consecutive 24-h food recalls conducted over an average period of one month. The mean number of days between the recalls was ten days. The method used for dietary assessment is described in detail elsewhere [20]. The diet quality score was developed based on healthfulness and micronutrient adequacy (Table 1). Similar scores have been used by our group in other settings [21,22]. Quartiles of DQS, ranging from 0 to 3, were used in the analyses (Table 2).

**Table 1:** Diet Quality Score (DQS) construction

Individual component	Score
Healthfulness (0–8 points)	
	Fat % energy 15–30% = 1; No = 0
	SFA % energy <10% = 1; No = 0
	PUFA % energy 6–10% = 1; No = 0
	Sugars % energy <10% = 1; No = 0
	Protein % energy 10–15% = 1; No = 0
	Cholesterol intake <300 mg/d = 1; No = 0
	Fibre intake >25 g/d = 1; No = 0
	Fruit and vegetable intake ≥400 g/d = 1; No = 0
Micronutrient adequacy (0–7 points)	
Vitamin A adequacy	
Vitamin E adequacy	
Vitamin C adequacy	
Thiamin adequacy	
Riboflavin adequacy	
Niacin adequacy	
Vitamin B_6 _adequacy	Micronutrient intake is ≥100% of recommended daily intake = 0.5; Micronutrient intake <100% of RDI = 0
Vitamin B_12 _adequacy	
Pantothenic acid adequacy	
Folate adequacy	
Magnesium adequacy	
Calcium adequacy	
Iron adequacy	
Zinc adequacy	
Diet Quality Score (0–15 points)	1^st ^quartile (0–8) = 0
	2^nd ^quartile (8.5–10) = 1
	3^rd ^quartile (10.5–11) = 2
	4^th ^quartile (11.5–15) = 3

SFA: Saturated fatty acids; PUFA: Polyunsaturated fatty acids

**Table 2:** Overall Lifestyle Score (OLS) construction

Individual component	Score	N (%)	Male (%)	Female (%)
Diet Quality Score (0–3)				
1^st ^quartile	0	55 (27.5)	19	36
2^nd ^quartile	1	61 (30.5)	31	30
3^rd ^quartile	2	47 (23.5)	27	20
4^th ^quartile	3	37 (18.5)	23	14
Smoking Score (0–3)				
Smokers ≥10 cigarettes/day	0	2 (1)	2	0
Smokers <10 cigarettes/day	1	3 (1.5)	3	0
Former smokers	2	18 (9)	17	1
Non smokers	3	177 (88.5)	78	99
Alcohol Consumption Score (0–3)				
Binge drinkers	0	36 (18)	13	23
Regular high drinkers	1	36 (18)	21	15
Non drinkers	2	76 (38)	29	47
Regular moderate drinkers	3	52 (26)	37	15
Physical Activity Score (0–3)				
Inactive (no physical activity)	0	41(20.5)	6	35
Light (moderate <30 min)	1	42 (21)	8	35
Moderate (moderate ≥30 min)	2	77 (38.5)	47	29
Active (vigorous ≥20 min)	3	40 (20)	39	1
Overall Lifestyle Score (0–12)				
1st tertile (Low)	0–6	61 (30.5)	14	48
2^nd ^tertile (Medium)	7–8	76 (38)	39	34
3rd tertile (High)	9–12	63 (31.5)	47	18

Healthfulness of diet: Eight WHO/FAO dietary recommendations for the prevention of the chronic diseases [23] were used to assess the healthfulness of the diet. These refer to total fat, saturated fatty acids, polyunsaturated fatty acids, cholesterol, sugar, protein, fruit and vegetable, and fibre. We did not use the recommendations on (n-3) and (n-6) fatty acids because these nutrients were not included in the database used. A score of 1 was given to each item if the recommendation was met and 0 if it was not, for a maximum total score of 8.

Micronutrient adequacy of diet: The adequacy of intake of 14 micronutrients (vitamins A, B_6_, B_12_, C and E, thiamin, riboflavin, niacin, pantothenic acid, folates, magnesium, calcium, iron and zinc) was checked against the recommended dietary intakes (RDI) for age and sex [24]. A score of 0.5 was given for 100% adequacy and above, and 0 if below 100%. The score of 0.5 was given to each item to reach a maximum total score of 7 for micronutrient adequacy, which is more or less equal to the maximum score assigned to healthfulness of diet.

#### Smoking score (SS)

Data on smoking were collected by pre-tested questionnaire. This questionnaire was also used to collect information on alcohol use, and on socio-economic as well as urbanization status. The smoking score was computed based on both actual smoking status and number of cigarettes smoked daily. Four categories were identified: current smokers ≥10 cigarettes/day, current smokers <10 cigarettes/day, former smokers and non smokers, with respective scores from 0 to 3 (Table 2). The score is actually a tobacco abstention score.

#### Alcohol consumption score (ACS)

Alcohol consumption was assessed by questioning the subjects about their habitual drinking patterns. Subjects were asked about the average daily amount of alcoholic beverages. A standard unit of one drink was used to assist respondents: 1 bottle of beer (33cl), 1 glass of wine (11cl) or 1 shot of distilled spirit (3.5cl). The alcohol consumption score was computed based on both the pattern of alcohol consumption (binge or regular) and the mean quantity of alcohol drunk daily. Four categories were identified: binge drinkers, regularly high, non drinkers and regularly moderate, with an ascending gradient of scores from 0 to 3 (Table 2). Moderate alcohol consumption is associated with a decreased CVD risk compared with total abstinence [25] and therefore, it was assigned the highest score. Four or more drinks on one occasion for women and five or more drinks for men were considered as binge drinking [11]. Moderate alcohol consumption was defined as up to two drinks per day for men and one drink per day for women [26].

#### Physical activity score (PAS)

Data on physical activity was collected with three 24-hour recalls of activities, based on the same technique as the 24-hour food recall. Subjects were asked to report all activities performed the day before each interview. A physical activity score was computed taking account of both the intensity (light, moderate or vigorous) and the duration of physical activity. The compendium of physical activity was used to assign the metabolic rate (in METs) for each activity [27]. Activities were grouped in three categories using the following classification [28]: low intensity activity (MET <3), moderate intensity activity (MET between 3 and 6) and high intensity activity (MET>6). The total number of hours for each level of activity was computed. Participants were grouped into four categories: inactive (engaging in no moderate or strenuous physical activities), light (participation in moderate-intensity activities for less than 30 minutes per day), moderate (participation in moderate-intensity activities for at least 30 minutes per day) and active (participation in high-intensity activities for more than 20 minutes per day), with corresponding scores of 0 to 3 (Table 2). Several sets of recommendations are in existence regarding physical activity. The most common recommendation is a minimum of 30 minutes of moderate-intensity physical activity each day [23,29]. It was also suggested that 20 minutes of vigorous-intensity activity each day was adequate [28]. Our scoring scheme for physical activity was derived from all these recommendations.

### Urbanisation status

Birthplace and length of city residence were used as proxy measures of urbanisation. Subjects were asked to name the place where they were born. Based on administrative data [14], we classified the locations as "urban" or "rural". Total duration of urban residence was divided in three groups on the basis of tertiles (≤20 years, 21–33 years and ≥34 years).

### Socioeconomic status

The SES score was based on education, occupation and household amenities. The score ranged from 0 to 6. The SES score was divided in three groups on the basis of tertiles (low, medium and high). Cronbach's alpha for the SES score was 0.71.

#### Education

Three education levels were considered: no schooling, primary school, and secondary school or above, with respective partial scores of 0, 1, and 2.

#### Occupation

Three categories of occupation were defined based on the scale used by the Benin National Institute of Statistics and Economic Analysis [14]. The first category (unskilled) was coded 0 and included the unemployed and workers engaged in occupations which generally require no special skills. The second category (semi-skilled) was coded 1 and the third category, coded 2, included skilled professionals and managers.

#### Household amenities

Household asset ownership was used as a proxy measure for income because in developing country settings it better reflects economic status than income [30]. Ten variables deemed appropriate for the Benin context were used: type of latrine, floor, roof, and sidewalls; type of fuel used for cooking; presence in the home of a paid domestic helper, electricity, television set, house phone, and fridge. A maximum score of 1 was assigned to each of these component variables. The first five variables had three levels so that a zero score referred to low, a score of 0.5 to intermediate and a score of 1 to high level. The last five variables were dichotomous and coded 0 for the absence and 1 for the presence of the amenity. The household amenity score was the sum of the individual scores, for a maximum of 10. On the basis of tertiles, low household amenity level was coded 0 (total score between 0 and 4), while medium (total score between 4.5 and 6.5) and high levels (total score between 7 and 10) were respectively coded 1 and 2.

### Statistical methods

Data were analysed using SPSS, version 13.0 (SPSS Inc, Chicago, IL). Differences between men and women were assessed using the two-tailed t-test. Prevalence rates of cardio-metabolic risk factors according to birthplace, length of urban residence and socioeconomic variables were compared using the chi-square test. Logistic regression analyses were used to determine the age-and-sex-adjusted odds ratio of CVD risk factors for each category of birthplace, length of urban residence and SES. Multiple linear regression analyses were performed to assess the associations between individual lifestyle factors and biological variables, while controlling for potential confounders such as sex, age and SES. Pearson's correlation coefficient was used to assess the relationships among lifestyle factors. Logistic regression was performed to assess the likelihood of obesity and cardio-metabolic risk factors according to the composite measure of lifestyle, the OLS. The level of statistical significance was a p value of <0.05 for all tests except regression models, for which the null hypothesis is rejected up to p < 0.1 [31].

### Ethical considerations

The study was approved by the Ethics Committee of the Faculty of Medicine, Université de Montréal, and by the Ministry of Health in Benin. The study objectives were clearly explained to participants, local authorities and the respective heads of selected households. Written informed consent was obtained from each participant before enrolment. The participants were all informed of their blood pressure and the results of laboratory tests. Those with abnormal values were referred to a physician for diagnosis and treatment.

## Results

A total of 200 subjects completed the study. This represents a 78 % participation rate; 25 subjects refused to take part in the study and another 33 subjects did not complete the study. Table 3 summarises the socio-economic characteristics of the subjects. Women were significantly less educated than men, which is reflected in a significantly lower SES. No difference was noted between men and women for the other socio-economic variables.

**Table 3:** Socioeconomic and demographic characteristics of participants

	All (n = 200)	Men (n = 100)	Women (n = 100)	P*
Age (years)	38.9 ± 9.8	37.8 ± 9.9	39.9 ± 9.6	0.119
Birthplace (%)				
Rural	35.5	35	36	0.500
Urban	64.5	65	64	
Length of urban residence (%)				
≤20 years	34	30	38	0.485
21–33 years	34.5	37	32	
≥34 years	31.5	33	30	
Education level (%)				
None	17	6	28	<0.001
Primary	29.5	24	35	
Secondary or higher	53.5	70	37	
Occupation (%)				
Unskilled	62	57	67	0.296
Semi-skilled	12.5	13	12	
Skilled	25.5	30	21	
Household amenities (%)				
Low	23.5	18	29	0.090
Medium	47	54	40	
High	29.5	28	31	
SES score	3.1 ± 1.9	3.5 ± 1.7	2.7 ± 1.9	0.002

* For difference between sex groups (two-sided t-test or χ^2^-test)

Values are expressed as mean ± standard deviation or percentages

SES score included education level, occupation and household amenities

Anthropometric and biological data are shown in Table 4. Mean BMI and waist circumference were markedly higher among women than men (p < 0.001). Serum HDL-cholesterol level was significantly lower among men compared to women (p < 0.001). In contrast, triglyceride concentrations were significantly higher in men compared to women (p < 0.001). Systolic and diastolic blood pressure did not vary significantly according to sex. Likewise, there was no sex difference in plasma glucose. The most prevalent risk factors in the study sample were overall obesity (18%), abdominal obesity (32%), hypertension (23%), and low HDL-cholesterol (13%). Diabetes and hypertriglyceridemia were uncommon. Women had a much higher rate of overall and abdominal obesity compared to men. One woman out of three was obese while over half had abdominal obesity according to international criteria. There was no statistically significant sex difference in the prevalence of hypertension and low HDL-cholesterol levels.

**Table 4:** Anthropometric and biological characteristics of the subjects and prevalence of CVD risk factors

	All (n = 200)	Men (n = 100)	Women (n = 100)	P*
Biological variables**				
BMI (kg/m^2^)	25.7 (5.6)	23.4 (4.4)	28.1 (5.8)	<0.001
WC (cm)	87.8 (13.3)	84.4 (12.7)	92.1 (13.1)	<0.001
TG (mmol/l)	0.8 (0.4)	0.9 (0.4)	0.7 (0.3)	0.005
HDL-chol (mmol/l)	1.3 (0.3)	1.2 (0.3)	1.3 (0.4)	0.002
SBP (mmHg)	124.4 (21.7)	121.9 (19.0)	126.9 (24.0)	0.103
DBP (mmHg)	73.8 (12.9)	72.0 (11.7)	75.5 (13.8)	0.054
FPG (mmol/l)	4.6 (0.6)	4.6 (0.6)	4.6 (0.5)	0.785
Prevalence of CVD risk factor***				
Overall obesity	18 (13.1–23.6)	8 (3.7–14.4)	28 (19.9–37.2)	<0.001
Abdominal obesity	32.5 (23.6–39.2)	11 (5.9–18.1)	54 (44.3–63.5)	<0.001
Hypertension	23 (17.5–29.1)	20 (13–28.5)	26 (18.1–35.1)	0.401
Hypertriglyceridemia	2 (1.0–4.6)	3 (0.7–7.7)	1 (0–4.6)	0.621
Low HDL cholesterol	13 (8.8–18.1)	10 (5.1–16.9)	16 (9.7–21.0)	0.293
Diabetes	0.5 (0.0–2.3)	1 (0–4.6)	0	

* For difference between sex groups (t-test or χ^2^-test).

**Values are expressed as mean (standard deviation)

*** Values are expressed as prevalence (95% CI)

**Figure 1:**
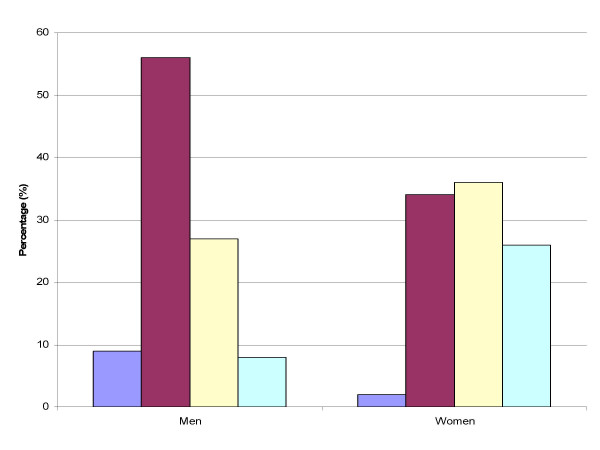
BMI distribution in the study sample. Purple bar: IMC < 18.5, Red bar: IMC 18.5-24.99, Yellow bar: IMC 15-29.99, Light blue bar: IMC ≥ 30

The nutritional status of the subjects according to BMI categories is shown in Figure 1. The overall prevalence of chronic energy deficiency (BMI<18.5) was 5.5%, while 31.5% of the subjects were overweight (BMI range 25–29.9). Chronic energy deficiency was higher in men compared to women, while an inverse trend was observed for overweight and obesity. Obesity exceeded chronic energy deficiency in women.

Age- and sex-adjusted odds ratios for obesity and other CVD risk factors according to SES and urbanisation status are shown in Table 5. The risk of obesity increased significantly with SES but not urbanisation, while a longer exposure to the urban environment but not SES was associated with a higher risk of hypertension.

**Table 5:** Age and sex-adjusted odds ratio (OR) for CVD risk factors by categories of SES and urbanisation status*

	Overall obesity		Abdominal obesity		Low HDL-cholesterol		Hypertension	
	
	OR (95% CI)	P	OR (95% CI)	p	OR (95% CI)	p	OR (95% CI)	P
SES								
Low (ref.)	1.0		1.0		1.0		1.0	
Medium	1.5 (0.5–4.7)	0.449	1.7 (0.7–4.2)	0.242	2.6 (0.9–8.2)	0.100	1.8 (0.7–4.0)	0.220
High	9.7 (3.0–20.9)	<0.001	2.6 (0.9–7.0)	0.066	1.7 (0.5–6.2)	0.439	1.4 (0.4–4.0)	0.586
Length of urban residence								
≤20 years (ref.)	1.0		1.0		1.0		1.0	
21–33 years	0.8 (0.3–2.1)	0.607	0.9 (0.4–2.2)	0.801	0.9 (0.3–2.7)	0.841	1.3 (1.1–6.9)	0.041
≥34 years	1.8 (0.7–5.1)	0.245	2.3 (0.9–5.7)	0.100	1.5 (0.5–4.3)	0.461	2.8 (1.1–6.9)	0.031
Birthplace								
Rural (ref.)	1.0		1.0		1.0		1.0	
Urban	0.8 (0.3–1.8)	0.542	1.4 (0.6–3.1)	0.410	0.9 (0.4–2.4)	0.893	0.8 (0.3–1.8)	0.533

* Logistic regression analysis was not performed for diabetes and hypertriglyceridemia because they were almost non-existent in the study sample.

Overall obesity (dependent variable): Hosmer and Lemshow test: χ^2 ^= 5.684; p = 0.683

Abdominal obesity (dependent variable): Hosmer and Lemshow test: χ^2 ^= 3.950; p = 0.862

Hypertension (dependent variable): Hosmer and Lemshow test: χ^2 ^= 4.316; p = 0.826

Low HDL-cholesterol (dependent variable): Hosmer and Lemshow test: χ^2 ^= 6.697; p = 0.570

The correlation matrix of lifestyle factors is given in Table 6. Alcohol consumption (ACS) was positively correlated with physical activity (PAS) in men (r = 0.24, p < 0.05) and DQS in women (r = 0.24, p < 0.05). Dietary quality (DQS), ACS and PAS were positively and significantly correlated with OLS in both sexes. ACS and DQS were more strongly correlated with OLS in women, while ACS and PAS were more strongly correlated with OLS in men. Smoking (SS) showed no significant association with the other lifestyle factors.

**Table 6:** Pearson's correlation coefficients (r) among lifestyle factors according to sex

Men/Women	DQS	SS	ACS	PAS	OLS
DQS	___	0.11	0.24*	-0.06	0.64***
SS	-0.10	___	0.15	-0.01	0.09
ACS	-0.13	-0.01	___	0.06	0.64***
PAS	0.07	-0.09	0.24*	___	0.40***
OLS	0.44***	0.17	0.59***	0.58***	___

*p < 0.05; **p < 0.01; ***p < 0.001

Values for women are at the upper right hand of the lines and for men at the lower left hand

DQS: Diet quality score; SS: Smoking score; ACS: Alcohol consumption score; OLS: Overall lifestyle score

The independent associations between lifestyle factors and biological variables are shown in Table 7. A higher PAS was associated with a significantly lower level of BMI (β = -0.43, p < 0.001), WC (β = -0.09, p = 0.036), systolic blood pressure (β = -0.23, p = 0.012) and diastolic blood pressure (β = -0.24, p = 0.010). PAS also tended to be negatively associated with fasting plasma glucose level (β = -0.15, p = 0.123). WC decreased significantly as ACS increased (β = -0.07, p = 0.028). DQS and SS did not show any significant association with biological variables.

**Table 7:** Multiple linear regression of biological variables on lifestyle factors

	BMI*		WC**		HDL-Chol**		TG**		SBP**		DBP**		FPG**	
	
	B	P	β	p	β	p	β	P	β	P	β	p	β	p
DQS	0.01	0.819	-0.01	0.662	-0.06	0.446	0.01	0.979	-0.03	0.612	-0.04	0.522	0.05	0.542
SS	-0.05	0.374	-0.06	0.050	-0.11	0.106	-0.12	0.077	-0.03	0.709	-0.09	0.187	0.02	0.840
ACS	-0.03	0.657	-0.07	0.028	0.04	0.558	-0.03	0.672	0.03	0.686	-0.03	0.693	0.05	0.468
PAS	-0.43	<0.001	-0.09	0.036	0.10	0.280	-0.05	0.549	-0.23	0.012	-0.24	0.010	-0.15	0.123

R^2^***	0.40		0.83		0.16		0.25		0.26		0.22		0.12	

* The model includes age, sex, SES, and lifestyle factors.

** The model includes age, sex, SES, BMI and lifestyle factors.

*** All of the models' R^2 ^were significant

DQS: Diet quality score; SS: Smoking score; ACS: Alcohol consumption score; PAS: Physical activity score BMI: Body mass index; WC: Waist circumference; HDL-chol: High density lipoprotein cholesterol; TG: Triglycerides; SBP: Systolic blood pressure; DBP: Diastolic blood pressure; FPG: Fasting plasma glucose.

The odds-ratios for obesity or other metabolic abnormalities according to global lifestyle score (OLS) are shown in Table 8. Compared with the lower OLS category, subjects in the upper OLS category had a significantly lower likelihood of overall obesity (OR = 0.07; CI, 0.02–0.29; p < 0.001), abdominal obesity (OR = 0.18; CI, 0.04–0.80; p = 0.025), and hypertension (OR = 0.19; CI, 0.05–0.68; p = 0.011). Likewise, subjects in the medium OLS category were at significantly lower odds of having overall obesity (OR = 0.35; CI, 0.13–0.90; p = 0.030) and abdominal obesity (OR = 0.24; CI, 0.05–1.12; p = 0.070) compared with subjects in the lower OLS category.

**Table 8:** Adjusted odds ratio (OR) for CVD risk factors according to lifestyle scores

OLS	Overall obesity*^1^		Abdominal obesity**^2^		Low HDL-cholesterol**^3^		Hypertension**^4^	
	
	OR (95% CI)	P	OR (95% CI)	p	OR (95% CI)	p	OR (95% CI)	P
Low (reference)	1.00		1.00		1.00		1.00	
Medium	0.35 (0.13–0.90)	0.030	0.24 (0.05–1.12)	0.070	0.98 (0.37–2.60)	0.974	0.68 (0.28–1.65)	0.396
High	0.07 (0.02–0.29)	<0.001	0.18 (0.04–0.80)	0.025	0.60 (0.17–2.12)	0.431	0.19 (0.05–0.68)	0.011

* The model included age, sex, SES and OLS

** The model included age, sex, SES, BMI and OLS

^1^Holsmer-Lemshow test: χ^2 ^= 7.138; p = 0.522

^2^Holsmer-Lemshow test: χ^2 ^= 6.494; p = 0.592

^3^Holsmer-Lemshow test: χ^2 ^= 5.393; p = 0.715

^4^Holsmer-Lemshow test: χ^2 ^= 5.027; p = 0.755

Logistic regression was not performed for diabetes and hypertriglyceridemia because they are almost non-existent in the study sample.

## Discussion

In this cross-sectional study, we assessed the prevalence of obesity and cardio-metabolic risk factors in apparently healthy urban adults in Benin and explored whether birthplace, length of urban residence and SES were associated with these factors, after controlling for age and sex. We also assessed the association of several modifiable lifestyle factors, taken individually or jointly, with these risk factors. We considered the cardio-metabolic risk factors individually rather that their clustering as metabolic syndrome because different definitions of the syndrome are used [32], and rates may vary considerably depending on the sets of criteria. Furthermore, the value of the metabolic syndrome as predictor of CVD beyond its component abnormalities remains controversial [33].

The observed high rate of obesity, particularly among women, is consistent with previous studies in urban Africa [34,35]. The influence of environmental, behavioural, psychosocial, and genetic factors on obesity is well recognised [36]. Sedentary lifestyles are common in urban women compared with men, as we verified using a novel approach consisting of 24-hour recalls of activities. Most women in our study were only involved in activities that are not physically demanding. The average score for physical activity was significantly lower in women compared to men and this may explain the difference in the prevalence of obesity between the two groups. Cultural values and the positive social attitudes towards fatness among women in Africa [37,38] are also conducive to feminine obesity as our study confirmed (data not shown).

Our findings show that the risk of obesity increased significantly with rising socioeconomic status. Subjects in the upper SES group have higher access to food and they may maintain a positive energy balance over a prolonged period of time, while periodic food shortage may be common among the poor [39]. Similar to our results, a positive relationship between SES and obesity has been reported in Cameroon [40]. Our findings suggest that the study population is still in the early stages of the nutrition transition since excess weight is currently seen primarily among the affluent, before progressively shifting to lower-income groups, as demonstrated in middle-income developing countries at later stages of the nutrition transition [41]. Based on national data, the burden of obesity appears to shift towards the poorer groups as the country's gross national product reaches the level of upper middle income countries [41]. However, these are aggregated data and the shift of the obesity burden towards the poor may be expected to take place even in low income countries, at least in large cities.

The fact that SES is more closely associated with overall obesity than abdominal obesity in multivariate analyses is an intriguing observation. The WHO cut-off levels for waist circumference or BMI as used in the present study may not be appropriate for African populations. Indeed, it is recommended to define specific BMI or waist circumference cut-offs for different race-ethnicity groups [42,43]. Race-ethnicity-specific WC cut-offs have been proposed in the USA [44] but these may not necessarily apply to Africans. Lower BMI cut-offs for overweight and obesity have been suggested as alternative public health action points for Asian populations [43], but relevant data are not available for Africans.

We observed a high prevalence of hypertension, in spite of the fact that previously diagnosed subjects were excluded from the study. A high prevalence of hypertension was also reported in other African countries [45,46]. Clearly, hypertension is a major public health problem in sub-Saharan Africa. Although evidence suggests that people of African origin are more susceptible to hypertension [13], both genetic and environmental factors are intertwined. Unlike for obesity, we did not find that socio-economic status was associated with hypertension. This suggests that hypertension may affect all segments of a population, even in the early stages of the nutrition transition. However, the results of this study indicate that a longer duration of urban residence, independent of age and sex, was associated with a higher risk of hypertension. This was also observed in a black population of the Cape Peninsula, South Africa [10]. Similarly, a positive rural-urban gradient for the prevalence of hypertension was observed in a population-based survey conducted in Tanzania [47]. Social deprivation, financial constraints and pressure associated with city living is suspected to add to the risk of hypertension [8]. Urbanisation also plays a role in the occurrence of hypertension through psycho-social stress as previously found in Tanzania [48]. However, we did not collect data on stress, which is difficult to measure. The psycho-social determinants of hypertension need further study in urban populations of Africa.

The low prevalence rate of diabetes and hypertriglyceridemia in the present study is noteworthy. A lower propensity to an adverse blood lipid profile in people of African origin was suggested in a previous extensive review on the issue [49]. Regarding diabetes, the rate was low even if subjects excluded because of previously diagnosed diabetes were taken into account. In fact, the prevalence of diabetes would have reached 2.5% if they had been included in the study (data not shown).

As urbanisation is associated with changes in diet and physical activity [8], being born in a city could be a potential risk factor for obesity and related metabolic abnormalities. Although we found that birthplace was significantly associated with dietary patterns in a previous study conducted in the same population [20], it did not show a significant association, however, either with obesity or with other metabolic risk factors in the present study. This is at variance with a study conducted among Mexican adults living in the United States, which showed that Mexican-born men and women had a lower risk of obesity than their U.S-born counterparts [50]. This suggests that the influence of early exposure to urban life on obesity or related risk factors may vary according to the context. However, as indicated earlier, the population under study is still probably in the early stages of the nutrition transition and it may be possible to find an influence of early life exposure to urban life on CVD risk factors in the future.

Single lifestyle behaviours were correlated with overall lifestyle score, with the exception of smoking. Lifestyle behaviours indeed tend to cluster together, as reported in China and the USA [11]. For example, men who were physically inactive were also more likely to drink heavily in the present study. However, we did not find any significant association between alcohol consumption and smoking, at variance with several studies reporting such an association [51,52]. This may be ascribed to the low prevalence of smoking in the study population (2.5%). Data on smoking status was based on self-reported information, typically collected via questionnaire, which may suffer from reliability problems. Probably one should determine biomarkers of tobacco smoking, i.e serum or plasma level of cotinine, the main metabolite of nicotine, to have more accurate information on smoking status [53].

Of the single lifestyle factors examined, physical activity was the most strongly associated with a lower likelihood of overall obesity, abdominal obesity and hypertension. This is in line with previous studies that assessed the association between lifestyle factors and metabolic abnormalities [54,55]. Our results showed that young subjects were more active than old ones. This is not surprising as the prevalence of most of the CVD risk factors increased with age (data not shown). We were not able to assess the effect of lifestyle behaviours on diabetes and hypertriglyceridemia because of the small number of cases.

Paradoxically, dietary quality did not show a significant association with obesity and other CVD risk factors, which is in contrast with the nutrition transition theory [3]. One possible explanation is that the population under study is still in the early stage of the nutrition transition and the diet is still low in fat and sugar, as previously reported [20]. The lack of association between diet and the risk of CVD could also be ascribed, at least partly, to the diet quality assessment method. The dietary scores that we developed and used in several settings [20-22] are the first to integrate the more recent WHO recommendations for the prevention of chronic diseases. Several diet quality indexes have been developed and subsequently modified and adapted [56]. However, only few showed a significant relationship with health risk [57]. Diet quality indexes still need to be used and interpreted with care as recently suggested because their development is based on empirical choices [56]. Location-specific food-based dietary guidelines are urgently required for culturally and economically relevant nutrition communication and as a basis for evaluating the quality of local dietary patterns.

Unlike Kim et al [11], we gave the same weight to all individual components of the lifestyle score, which assumed that they contributed equally to the score. However, ideally, the weighting of risk factors should be determined based on extensive data on the relative health risk associated with each lifestyle component in longitudinal studies. Unfortunately, such data do not exist for African populations. We found that the likelihood of overall obesity, abdominal obesity, and hypertension decreased significantly as the overall lifestyle score improved. These results suggest that in this population, the majority of cases of obesity and other related metabolic abnormalities could be curbed by the adoption of healthier lifestyles, particularly as regards physical activity.

To our knowledge, there is no universal definition of urbanisation status. Proxy measures such as birthplace and length of urban residence were used to measure urbanisation status in this study. Length of urban residence was recorded by summing the time lived in a city, from birth until the time of data collection. A similar approach, but using a more detailed questionnaire, was used in a previous study conducted in Cameroon [9]. Birthplace was used because it is closely related to acculturation [58].

It has been suggested that multiple indicators of SES should be used when assessing the influence of socioeconomic factors on health [59]. This issue was addressed in the present study because our SES score was computed based on three different sets of indicators – education, main occupation, and household assets. However, even studies that include multiple standard SES measures may be fall of potentially important socioeconomic influence on health [56]. It is clear that some unmeasured socioeconomic factors may have affected obesity or other CVD risk factors in our study population. Economic status impinges on health outcomes. In affluent societies, income is the most commonly used measure of economic status, while its measure poses a significant problem in developing countries because household income or expenditure levels are often unavailable or unreliable [60]. We did not use income as a direct measure of economic status because a large part of the population engages in informal work. As indicated by Houweling et al [30], expressing income in monetary value in countries where a large part of the population work in self-subsistence agriculture or the informal sector can be extremely time-consuming and suffers important reliability problems. Household assets are a better reflection of economic status than income in developing country settings, and were used as a proxy measure of income. Education was included in the SES score as it is related to health. Education includes several aspects, and years of formal education was used in the present study because of its potential effect on health [61].

Our study has several limitations. First, the cross-sectional design does not allow any inference to be drawn with respect to the causal relationship among variables. Second, the study sample is only representative of urban adults of Cotonou, and thus findings may not apply to the whole urban population of Benin. The study may probably lack statistical power due to the modest sample size. The conclusions of the study should therefore be interpreted with caution. These limitations notwithstanding, our study provides useful data on the prevalence of obesity and other CVD risk factors among adults of Cotonou, and their socioeconomic and lifestyle correlates.

## Conclusion

Our data show that CVD risk factors are highly prevalent among urban dwellers in Benin. This situation calls for preventive action to avert the rise of diet-related chronic diseases. People with higher SES and those having lived longer in the city are priority target groups at this time for action on obesity and metabolic abnormalities in this population. Our data also underline the relevance of lifestyle measures when designing public health actions against obesity and hypertension. Multiple-behaviour interventions would appear appropriate, with particular emphasis on physical activity.

## Competing interests

The author(s) declare that they have no competing interests.

## Authors' contributions

HD designed the study. RS collected the data under supervision of HD, BF and VA. RS and HD analysed the data and wrote the first draft of the manuscript. All the coauthors contributed to the revision and the finalisation of the paper.

## Pre-publication history

The pre-publication history for this paper can be accessed here:


